# Evaluation of 4‐Hz log files and secondary Monte Carlo dose calculation as patient‐specific quality assurance for VMAT prostate plans

**DOI:** 10.1002/acm2.13315

**Published:** 2021-06-20

**Authors:** Philipp Szeverinski, Matthias Kowatsch, Thomas Künzler, Marco Meinschad, Patrick Clemens, Alexander F. DeVries

**Affiliations:** ^1^ Institute of Medical Physics Academic Teaching Hospital Feldkirch Feldkirch Austria; ^2^ Private University in the Principality of Liechtenstein Triesen Liechtenstein; ^3^ Department of Radio‐Oncology Academic Teaching Hospital Feldkirch Feldkirch Austria

**Keywords:** log file, Monaco, SciMoCa, VMAT quality assurance

## Abstract

**Purpose:**

In this study, 4‐Hz log files were evaluated with an independent secondary Monte Carlo dose calculation algorithm to reduce the workload for patient‐specific quality assurance (QA) in clinical routine.

**Materials and Methods:**

A total of 30 randomly selected clinical prostate VMAT plans were included. The used treatment planning system (TPS) was Monaco (Elekta, Crawley), and the secondary dose calculation software was SciMoCa (Scientific‐RT, Munich). Monaco and SciMoCa work with a Monte Carlo algorithm. A plausibility check of Monaco and SciMoCa was performed using an ionization chamber in the BodyPhantom (BP). First, the original Monaco RT plans were verified with SciMoCa (pretreatment QA). Second, the corresponding 4‐Hz log files were converted into RT log file plans and sent to SciMoCa as on‐treatment QA. MLC shift errors were introduced for one prostate plan to determine the sensitivity of on‐treatment QA. For pretreatment and on‐treatment QA, a gamma analysis (2%/1mm/20%) was performed and dosimetric values of PTV and OARs were ascertained in SciMoCa.

**Results:**

Plausibility check of TPS Monaco vs. BP measurement and SciMoCa vs. BP measurement showed valid accuracy for clinical VMAT QA. Using SciMoCa, there was no significant difference in PTV Dmean between RT plan and RT log file plan. Between pretreatment and on‐treatment QA, PTV metrics, femur right and left showed no significant dosimetric differences as opposed to OARs rectum and bladder. The overall gamma passing rate (GPR) ranged from 96.10% to 100% in pretreatment QA and from 93.50% to 99.80% in on‐treatment QA. MLC shift errors were identified for deviations larger than −0.50 mm and +0.75 mm using overall gamma criterion and PTV Dmean.

**Conclusion:**

SciMoCa calculations of Monaco RT plans and RT log file plans are in excellent agreement to each other. Therefore, 4‐Hz log files and SciMoCa can replace labor‐intensive phantom‐based measurements as patient‐specific QA.

## 1 INTRODUCTION

In modern radiotherapy techniques, such as volumetric‐modulated arc therapy (VMAT), the gantry of the linear accelerator (linac) continuously rotates around the patient while delivering dose.^1^, ^2^ VMAT has many advantages compared to conventional radiation techniques, such as improved coverage of the target planning volume (PTV) and minimizing the dose at organs at risks (OARs).^3^ For VMAT plans, many different parameters of the linac have to be simultaneously and precisely coordinated to each other (e.g., MLC leaf positions, monitor units [MU], dose rate, gantry angle, collimator angle, jaw position, and energy). Due to the complexity of this radiation technique, consistent quality assurance (QA) for treatment delivery is required. Patient QA guarantees that the patient's correct dosimetry and safety is given. This includes verifying transfer from the treatment planning system (TPS) to the treatment machine and delivery of the calculated dose from the TPS.^4^

In general, QA systems can be classified as measurement‐based methods (e.g., ionization chamber, 2D and 3D arrays, radiochromic films, EPID) or as simulation‐based methods (e.g., log file analysis). Numerous studies investigate the sensitivity of phantom‐based patient‐specific QA with induced errors.^5^, ^6^, ^7^, ^8^, ^9^, ^10^, ^11^, ^12^, ^13^, ^14^ Other studies demonstrate that measurement devices are inferior to simulation‐based systems in detecting errors.^15^, ^16^, ^17^ Moreover, using log files for QA makes it possible to automatically check all deliveries in time without the need of a physical phantom and does not require access to the treatment machine. Log file analysis is a very time‐saving tool for patient QA in clinical routine. Because of these advantages, there is a growing interest in using log files with an independent Monte Carlo (MC) algorithm.^18^ Haga et al. investigated log file analysis for a single simple prostate plan and demonstrated that Elekta 4‐Hz log files have satisfying results for QA.^19^ Furthermore, Katsuta et al. showed a strong correlation between log file dose (using MC) and ionization chamber dose (physical measurement).^20^ Sun et al. could prove that an independent dose calculation algorithm (convolution superposition algorithm) with log file analysis is a reliable tool for IMRT (intensity‐modulated arc therapy) QA.^21^

In general, before and during treatment, log file analysis enables us to detect errors that occur when plan data are transferred from the TPS to the linac as well as dose calculation errors or beam delivery errors of the treatment machine. However, research has raised concern about the safety of only using log file analysis for patient QA without any other conventional measurements.^16^, ^22^ Hence, it is necessary to investigate accuracy and limits of log file analysis, for example, by comparing measurement‐based methods with log file analysis.^17^ Ce Han et al. performed a cross verification between measurement‐based ArcCHECK QA and simulation‐based log file QA using Elekta log files, and it was concluded that sensitivity for log file QA using a collapsed cone (cc) calculation algorithm was superior.^23^ In our study, an MC algorithm instead of a cc calculation algorithm was applied to use log files.

Several studies have already dealt with high resolution log files using Varian linear accelerators (Varian Medical Systems, Palo Alto).^24^, ^25^, ^26^, ^27^, ^28^, ^29^ Wei Luo et al. demonstrated that Monte Carlo simulation using Dynalog log files (recorded every 50 ms, 20 Hz) has numerous advantages in patient‐specific QA compared to measurement‐based QA.^24^ In measurement‐based QA, measurement uncertainties (using film or ionization chamber) might occur, only pretreatment QA is feasible and the information of the measurement in a phantom does not allow drawing conclusions about the dosimetric impact in the patient. However, using Dynalog log files with a Monte Carlo algorithm enables verifying leaf sequencing, data transfer, and beam delivery. Schreibmann et al. also showed that Dynalog log files are a convenient and practical way for dose reconstruction without the need of phantom measurements or phantom calculations.^25^ Wei Luo et al. stated that the high‐resolution log files used in their study are only available for Varian users.^24^ Teke et al. observed the accuracy and flexibility using Dynalog log files and found the sampling rate of 20 Hz to be sufficient.^26^

In this study, 4‐Hz Elekta log files are examined to determine their suitability for patient‐specific QA. Sampling rate and dynamic tolerances of the linac are decisive factors when using log files. For Elekta linear accelerators, dose rate is the leading parameter and all other dynamic parameters (e.g., leaf position, gantry position, and collimator position) must be within the tolerances. Acceleration and deceleration of leaves can lead to small deviations of a few millimeters within the dynamic tolerances of the linac. It is necessary to examine whether these small deviations have an influence on dose recalculation of 4‐Hz log file.

Recently, Piffer et al. first compared SciMoCa with TPS Monaco by means of treatment plans and dose measurements.^30^ They concluded that the MC‐based secondary dose calculation algorithm used in SciMoCa is a promising tool for pretreatment patient‐specific QA. In our study, the investigation of 4‐Hz log files was added to further enable on‐treatment delivery QA.

Our idea is combining all findings of previously mentioned studies for simple, non‐labor‐intensive and time‐saving patient‐specific QA. Log files with a gold standard independent secondary dose calculation algorithm are used to find any kind of errors, including dose calculation errors, transfer errors, and delivery errors. The secondary MC algorithm is first used for plausibility check (TPS dose data) and after irradiation for log file check. This check includes data transfer to the linac and delivery of the linac.

The aim of this study was to evaluate 4‐Hz log files with an independent secondary MC dose calculation algorithm to reduce the workload for patient‐specific quality assurance in clinical routine to guarantee patient safety. Combining and evaluating an independent secondary MC dose algorithm with 4‐Hz log files (Elekta) has, to the authors' knowledge, not been done before.

## 2 MATERIALS AND METHODS

### 2.1 Plan selection and treatment planning

A total of 30 randomly selected clinical prostate VMAT plans were included in this study. All plans were calculated with Monaco 5.11.02 (Elekta, Crawley) using Monte Carlo algorithm. The energy was set to 6 MV. Treatment plans were created using one beam and dual‐arc VMAT technique with a fraction of 2 Gy. The gantry range was −180° to +180°. The collimator angle was fixed during radiation between 0° and 45° depending on the plan. The minimum leaf gap was between 0.5 and 1.0 cm. Dose grid resolution varied between 0.25 and 0.30 cm with a statistical uncertainty of 0.5%–1% per plan. All dose calculations were performed applying dose‐to‐medium setting. These plans were accepted for clinical application and were delivered using three matched Elekta Synergy linear accelerators equipped with an Agility head (160‐leaf multileaf collimator, leaf‐width at isocenter 5 mm) and the Mosaiq 2.8.1 record and verify system (Elekta, Crawley). The PTV size of all 30 plans varied between 61 and 630 ccm.

### 2.2 Secondary dose calculation software—SciMoCa

For independent secondary dose calculation, SciMoCa 1.5 (Scientific‐RT, Munich) was applied to verify all prostate VMAT plans calculated by Monaco TPS. SciMoCa Software uses CT data, RT plan file, RT structure file and RT dose file from TPS to reconstruct a 3D dose distribution. Commissioning of SciMoCa with a Monte Carlo algorithm^31^, ^32^ was fully independent from Monaco and described in subsection (2.7). Therefore, changes in base data or any other dose calculation failure in clinical routine are immediately detected by SciMoCa. In this study, the statistical uncertainty was set to “fine” (computational uncertainty of 1%) for pretreatment QA and on‐treatment QA. For verificiaton of TPS Monaco and SciMoCa the computational uncertainty was set to “extra fine” (computational uncertainty of 0.5%). Dose grid resolution was set to 0.3 cm and External Density Threshold was activated at a limit of 0.1 g/cm³ for all calculations performed with SciMoCa. Also for SciMoCa, dose‐to‐medium setting was used for all calculations.

### 2.3 Cross verification of TPS Monaco and SciMoCa

All plans were measured with a 0.125‐ccm ionization chamber in a homogeneous phantom for simple cross verification of TPS Monaco and the independent secondary dose calculation software. These measurements were performed at isocenter using the BodyPhantom (BP) by IBA (IBA, Schwarzenbruck) and were compared with calculations from both systems, as a simple dosimetric check. This single‐point measurement method allows verification of the absolute dose at a certain point in the used BodyPhantom. According to AAPM Task Group No. 218 and the NCS report 24, point measurements are sufficient for patient‐specific QA if the validation process was clinically acceptable and if machine specific QA ensures that the linac has not changed since initial tests.^4^, ^33^

### 2.4 Plan delivery and log file processing

The treatment delivery of an Elekta linac is controlled by the treatment control system (TCS). It dynamically adjusts the linac parameters to deliver a treatment plan. The TCS works at a frequency of 25 Hz and creates a log file, which is not directly accessible. The linac provides the same data at a rate of 4‐Hz at the iCom Interface, which is accessed by LINACwatch® (Qualiformed, France). Kowatsch et al. showed that the difference between 25‐Hz and 4‐Hz log files is negligible for dose calculations.^34^ All log files contain several dynamic parameters such as leaf position, gantry position, collimator position and delivered monitor units. For reading and converting log files, the software LINACwatch was used. All log files had to be converted into DICOM RT plan files and were then referred to as RT log file plans, which were generated with as many control points as in the log file.

### 2.5 Pretreatment and on‐treatment patient‐specific QA

**Fig. 1:**
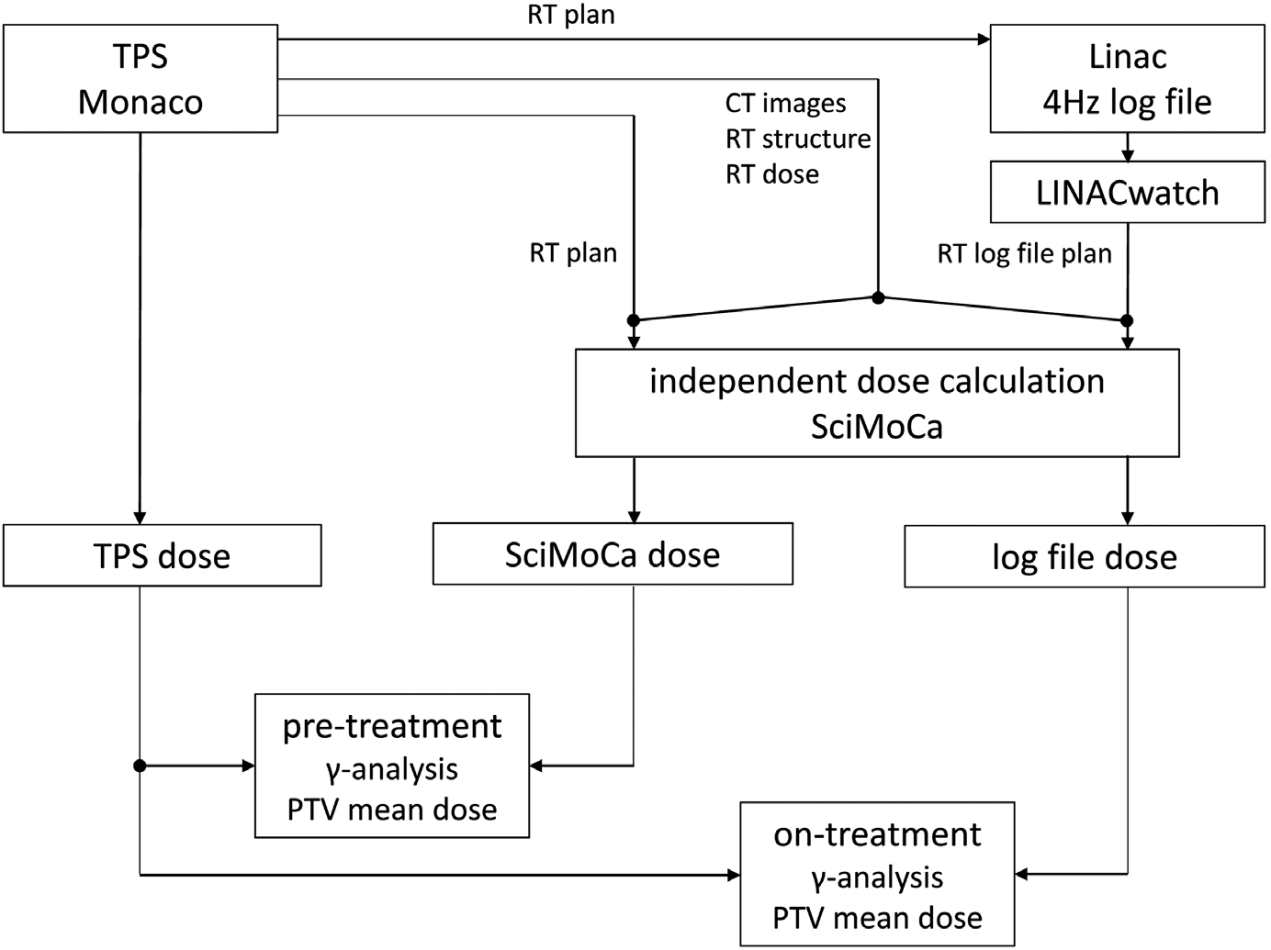
Flow chart of the entire pretreatment and on‐treatment QA used in this study. Pretreatment QA uses the RT plan of TPS Monaco and compares its dose distribution calculated in Monaco vs. SciMoCa using original CT images of the patient. In on‐treatment QA, the RT plan is delivered by the linac and then converted into an RT log file plan by the software LINACwatch. On‐treatment compares the TPS dose distribution of the RT plan with the dose distribution of the RT log file plan calculated with SciMoCa.

Patient‐specific QA was subdivided into pretreatment and on‐treatment QA. Both methods are fundamental for reliable patient‐specific QA to guarentee treatment safety of the patient. Patient‐specific QA is described in Fig. 1 as well as in Sections 2.5.1 and 2.5.2.

#### 2.5.1 Pretreatment patient‐specific QA

As pretreatment patient‐specific QA, the TPS Monaco dose distribution was checked with SciMoCa. All plan data (CT images, RT plan, RT structure and RT dose) were transferred from TPS Monaco to SciMoCa.

#### 2.5.2 On‐treatment patient‐specific QA

First, the RT plan was delivered by the linac and then converted into an RT log file plan by the software LINACwatch. This RT log file plan and the original CT images, RT structures and RT dose from TPS Monaco were used for calculations in SciMoCa. The calculated dose from SciMoCa was compared with the original TPS Monaco dose calculation. Hence, patient‐specific on‐treatment QA involves checking TPS calculation, data transfer to the linac, and delivery/treatment itself.

#### 2.5.3 Analysis of pretreatment and on‐treatment calculations

All calculations with SciMoCa were automatically performed within about 5 min. After these independent dose calculations, the gamma passing rates^10^ (GPRs), dosimetric values of PTV and OARs, and all other dose‐volume histogram (DVH) parameters were ascertained in SciMoCa. To compare dose distributions for pretreatment and on‐treatment, gamma analysis (2%/1mm/20%) was performed. For comparison of PTV and OARs, different Dx ([Gy], dose irradiated to x% of the target volume), different Vx ([%], volume irradiated to x% of the prescription dose) and individual volume GPRs (γVolume) were calculated. For PTV comparison Dmean, V95, V107, D2, D98 and γPTV were extracted. The most important OARs for prostate plans are bladder, rectum, femur left and femur right. For all OARs, numerous Dx and γVolumes were analyzed.

### 2.6 Sensitivity of MLC errors

To verify that error induced plans can be detected as such, one prostate plan was manipulated with an in‐house Matlab® (MathWorks Inc., Natick) tool. MLC misalignments with different magnitudes were applied (MLC opening and MLC closing from 0.25 to 0.75 mm, increment 0.25 mm). All six error induced plans were delivered by the linac and the corresponding log file was compared with the reference plan from TPS. For all error induced plans an on‐treatment QA was performed.

A gamma criterion of 2%/1 mm/20% (threshold) and a pass limit of 90% for pretreatment and on‐treatment QA for overall gamma was used. Moreover, PTV Dmean was set to 2% dose tolerance and a visual comparison of PTV coverage and OARs in DVH was performed by an experienced physicist.

### 2.7 Commissioning and validation of Monaco and SciMoCa

TPS Monaco beam data were collected applying Elekta guidelines and validated in 2013. The commissioning process for the XVMC dose algorithm was done by Elekta. Implementing MC‐based systems into clinical routine is well described in literature.^35^, ^36^

**Fig. 2:**
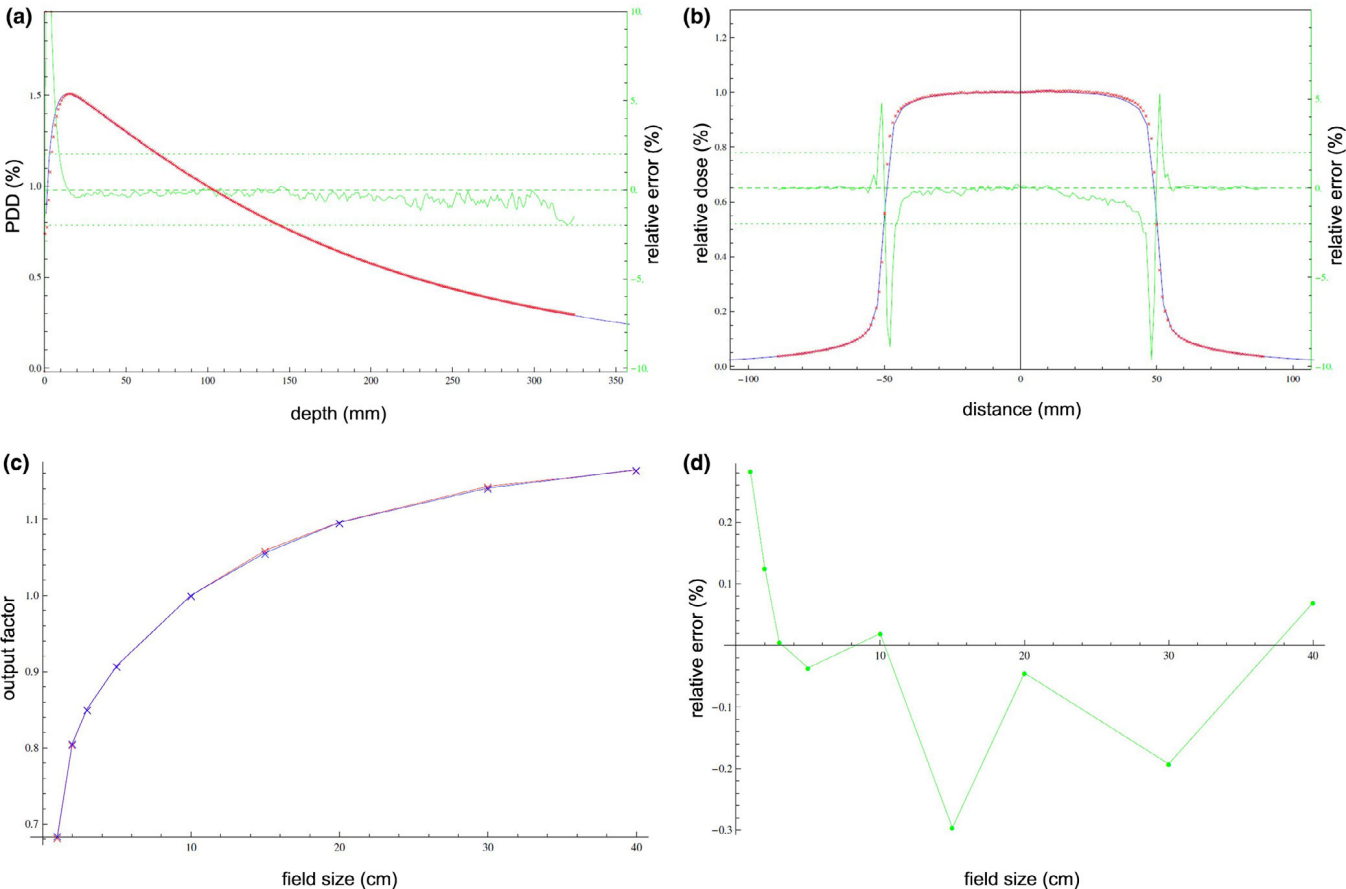
Measured values (red crosses); simulated values with SciMoCa (blue curve); green line (secondary y‐axis for Fig. 2(a) and 2(b), y‐axis for Fig. 2(d)) describes relative differences between measurement and simulation. (a) percentage depth dose curve (PDD) for 10 cm × 10 cm field size. (b) Inplane profile in 10 cm depth for 10 cm × 10 cm field size. (c) Output factors. (d) Relative error of output factors.

The introduced software SciMoCa uses a more precise Monte Carlo algorithm, which has already been reported in literature.^31^, ^32^, ^37^ Hoffmann et al. described the main parts of the SciMoCa algorithm.^37^ In 2018, our department decided to independently implement SciMoCa for pretreatment QA. Therefore, a set of measurements was done using different measuring devices. For the internal validation process of the vendor (Scientific‐RT, Munich) a set of measurements was needed to compare and adjust the beam model to the linac.^6^, ^7^, ^8^ All required measurements were performed three times and averaged for the adjustment of the SciMoCa model. The verification process of the vendor showed good agreement between simulation and measurement. A part of this process can be seen in Fig. 2.

The set of measurements, which was sent to the vendor, contained output factors and profiles for different field sizes and different depths as well as several depth dose curves. All measurements for output factors, profiles, and depth dose curves for small fields from 1 cm × 1 cm up to 10 cm × 10 cm were performed using microDiamond Type60019 (PTW, Freiburg). The output factors for fields from 15 cm × 15 cm up to 40 cm × 40 cm were measured using a farmer chamber PTW30013 (PTW, Freiburg). Profiles and depth dose curves for fields from 15 cm × 15 cm up to 40 cm × 40 cm were measured using a CC13 chamber (IBA, Schwarzenbruck).

### 2.8 Statistical analysis

Statistical analyses were performed using SPSS 23 (IBM, New York). Mean dose results and GPRs are presented as mean ± 1 SD. A *p* value smaller than 0.05 was defined as statistically significant and all *p* values are two‐sided. The analysis of correlation coefficient *r* was done according to Pearson. The Pearson correlation coefficient *r* was defined as weak for *r* < 0.4, moderate for *r* = 0.4–0.7 and strong for *r* > 0.7.

## 3 RESULTS

### 3.1 Cross verification of TPS Monaco and SciMoCa

**Fig. 3:**
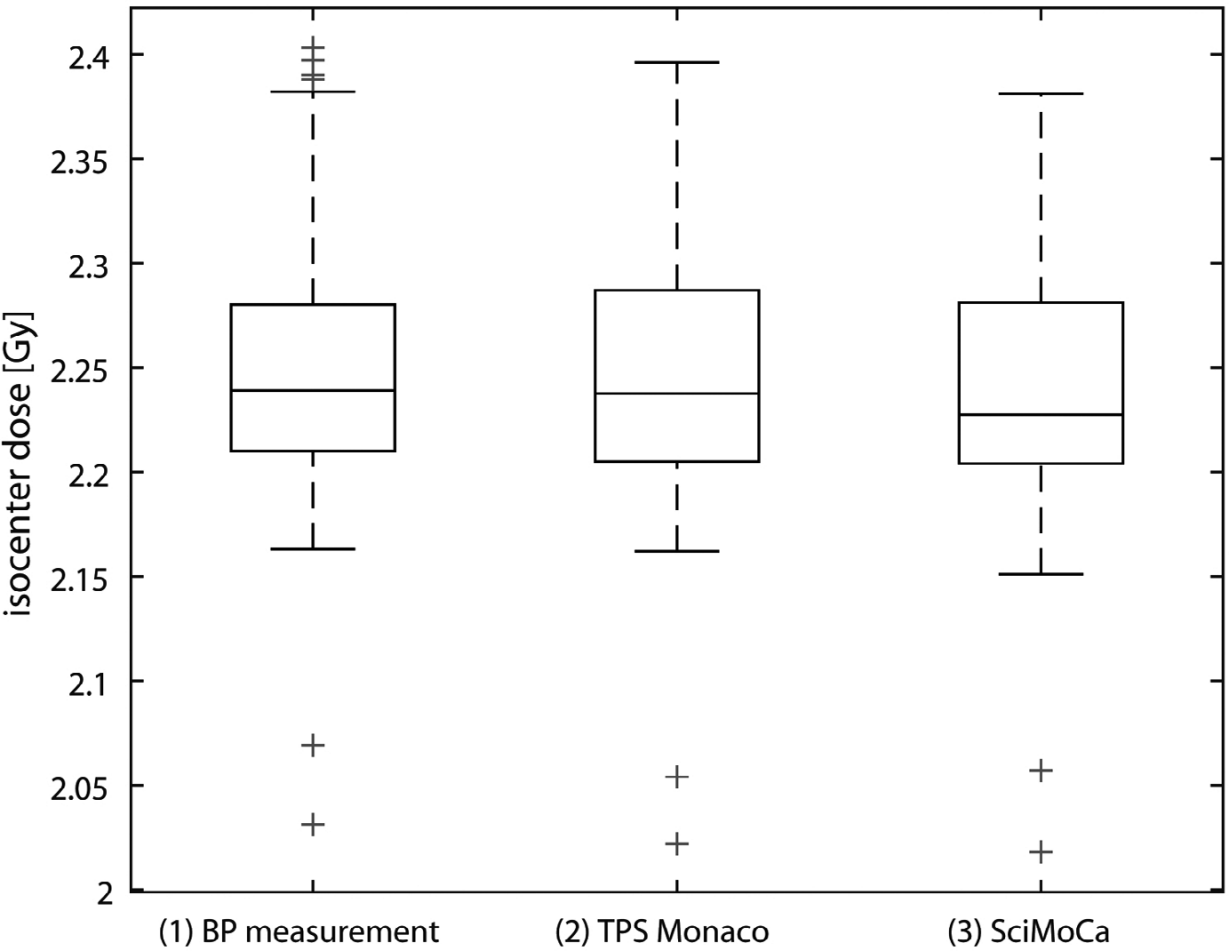
Boxplot of isocenter dose for (1) measurement with ionization chamber in the BodyPhantom (BP), (2) TPS Monaco calculation, and (3) SciMoCa calculation.

For all 30 prostate plans, the dosimetric difference between TPS Monaco vs. BP measurement and SciMoCa vs. BP measurement was −0.17 ± 0.43% (*p* = 0.06) and −0.45 ± 0.39% (*p* < 0.001), respectively. The deviation between TPS Monaco vs. BP measurement and SciMoCa vs. BP measurement showed valid accuracy for clinical VMAT QA. Dosimetric differences between TPS Monaco vs. SciMoCa was 0.29 ± 0.30% (*p* < 0.001). The maximum deviation between TPS Monaco vs. SciMoCa dose calculation at isocenter in the BP ranged from −0.30% to 0.69% for all plans. Figure 3 shows the measured dose compared to the calculated dose for TPS Monaco and SciMoCa.

**Fig. 4:**
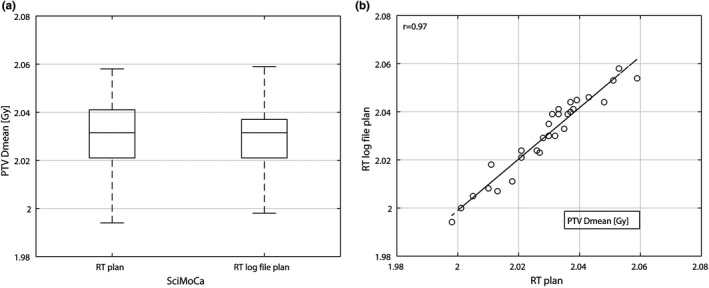
(a) Boxplot and (b) Scatterplot of PTV Dmean for recalculation of RT plan in SciMoCa and recalculation of RT log file plan in SciMoCa.

Target planning volume Dmean was compared between RT plan and RT log file plan in SciMoCa, which can be seen in Fig. 4. For RT plan calculation, the PTV Dmean was 2.03 ± 0.01 Gy and for RT log file plan 2.03 ± 0.02 Gy (*p* = 0.21). There was a strong correlation for PTV Dmean between RT plan and RT log file plan with *r* = 0.97 (*p* < 0.001).

### 3.2 Pretreatment and on‐treatment patient‐specific QA

Detailed dosimetric differences for different metrics for all prostate plans in pretreatment and on‐treatment patient‐specific QA are shown in Table 1. In all PTV metrics, in femur right and in femur left, no significant dosimetric differences were observed between pretreatment and on‐treatment QA. For the OARs rectum and bladder significant dosimetric differences occurred between pretreatment and on‐treatment QA.

**Table 1:** Dosimetric differences [%] in pretreatment and in on‐treatment QA for different metrics for all 30 prostate VMAT plans.

	Dosimetric differences [%]		
Metrics	Pretreatment QA	On‐treatment QA	*p*
PTV			
Dmean	0.41 ± 0.35	0.36 ± 0.43	0.24
V95	0.29 ± 0.48	0.32 ± 0.56	0.37
V107	0.07 ± 0.33	0.06 ± 0.45	0.12
D2	0.19 ± 0.38	0.51 ± 0.64	0.13
D98	0.55 ± 0.65	0.11 ± 0.49	0.64
Rectum			
D15	1.27 ± 1.53	0.94 ± 1.58	0.001
D20	1.52 ± 1.75	1.11 ± 1.76	<0.001
D25	1.57 ± 1.82	0.99 ± 1.93	<0.001
D35	1.70 ± 2.29	0.99 ± 2.33	<0.001
D50	1.93 ± 4.18	1.22 ± 4.36	<0.001
Bladder			
D15	−0.11 ± 1.45	−0.22 ± 1.71	0.18
D25	0.34 ± 2.00	0.18 ± 2.32	0.41
D35	1.74 ± 2.72	1.49 ± 2.59	0.02
D50	3.86 ± 3.42	3.26 ± 3.24	<0.001
Dmax	0.08 ± 0.58	−0.17 ± 0.80	0.06
Femur right			
Dmax	0.54 ± 0.91	0.69 ± 1.41	0.56
Femur left			
Dmax	0.92 ± 1.31	0.71 ± 1.52	0.64

Table 2 shows detailed GPRs for pretreatment and for on‐treatment patient‐specific QA in different γVolumes. The overall GPR ranged from 96.10% to 100% in pretreatment QA and from 93.50% to 99.80% in on‐treatment QA.

**Table 2:** Gamma passing rates (GPRs) [%] in pretreatment and in on‐treatment QA for different metrics for all 30 prostate VMAT plans.

γVolumes	GPRs [%]	
	pretreatment QA	on‐treatment QA
γoverall	99.4 ± 0.9	97.7 ± 1.8
γPTV	98.2 ± 2.6	96.9 ± 3.4
γRectum	98.1 ± 5.2	97.6 ± 5.9
γBladder	99.3 ± 1.4	98.9 ± 1.3

Note

The gamma criterion was set to 2% / 1mm / 20% and individual organs were evaluated (overall, PTV, rectum and bladder).

### 3.3 Sensitivity of MLC errors

One prostate plan was manipulated to see if the described pretreatment and on‐treatment patient‐specific QA programs can identify these errors.

**Fig. 5:**
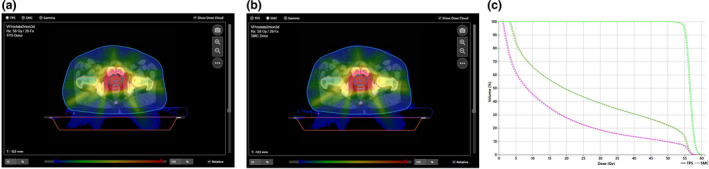
Pretreatment QA of the reference plan. (a) TPS dose distribution. (b) SciMoCa dose distribution (RT plan). (c) DVH analysis between TPS and SciMoCa dose distribution (dark green line: bladder, purple line: rectum, light green line: PTV).

First, the reference plan (error‐free plan) was analyzed in pretreatment QA, where the TPS dose distribution was compared to the SciMoCa dose distribution (see Fig. 5). The overall gamma passing rate was 98.9%, γPTV was 96.9% and PTV Dmean difference was 0.49%.

**Fig. 6:**
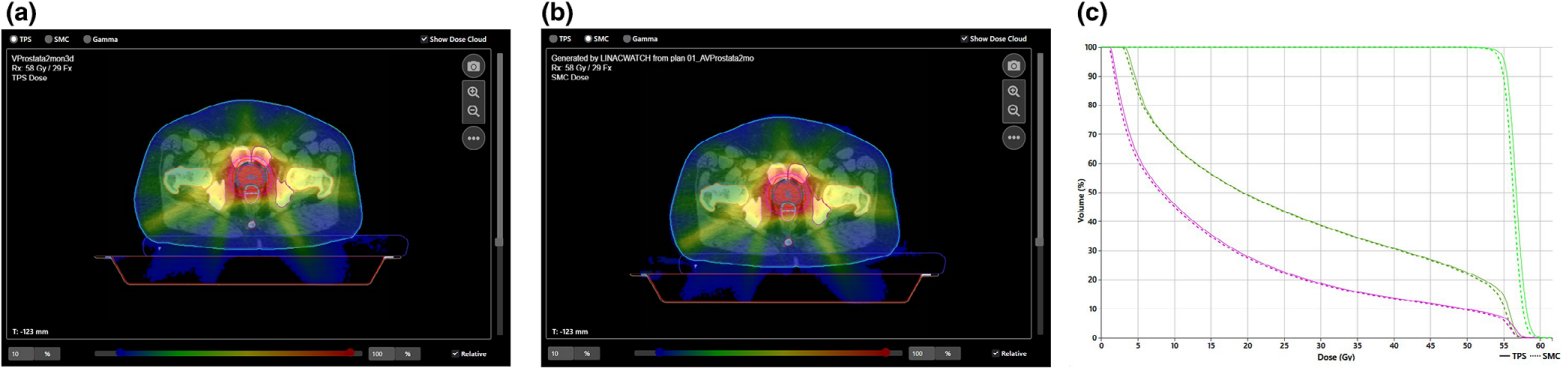
On‐treatment QA of the reference plan. (a) TPS dose distribution. (b) SciMoCa dose distribution (RT log file plan). (c) DVH analysis between TPS and SciMoCa dose distribution (dark green line: bladder, purple line: rectum, light green line: PTV).

Second, the reference plan and all error induced plans were analyzed in on‐treatment QA, where TPS dose distribution of the reference plan was compared to the SciMoCa dose distribution of the error induced RT log file plan. In on‐treatment QA using the reference RT log file plan, the overall gamma value was 96.8%, γPTV was 91.4% and PTV Dmean difference (∆PTV Dmean) was 0.81% (see Fig. 6).

In on‐treatment QA, the gamma value decreased when MLC shift error increased for all error induced RT log file plans. Furthermore, the impact of MLC shift error in PTV Dmean dose increased for enlarged field size and decreased for reduced field size. The clinical impact of MLC shift error was directly seen in the patient's dose distribution in SciMoCa. All error induced on‐treatment gamma values and ∆PTV Dmean values are in Table 3.

**Table 3:** Influence of MLC shift error in on‐treatment QA on γoverall, γPTV and ∆PTV Dmean.

MLC shift error	On‐treatment QA		
	γoverall[%]	γPTV[%]	∆PTV Dmean[%]
−0.75 mm	69.7	8.7	3.74
−0.50 mm	82.5	32.0	2.74
−0.25 mm	93.4	75.5	1.52
error‐free plan	96.8	91.4	0.81
+0.25 mm	97.9	96.3	−0.43
+0.50 mm	93.3	77.8	−1.48
+0.75 mm	87.5	50.3	−2.33

On‐treatment QA for error induced prostate plans identified MLC shift errors for deviations larger than −0.50 mm and +0.75 mm with the limits set to 2%/1 mm/20% (threshold) for overall gamma criterion, a pass limit of 90% and 2% for PTV Dmean difference (∆PTV Dmean).

**Fig. 7:**
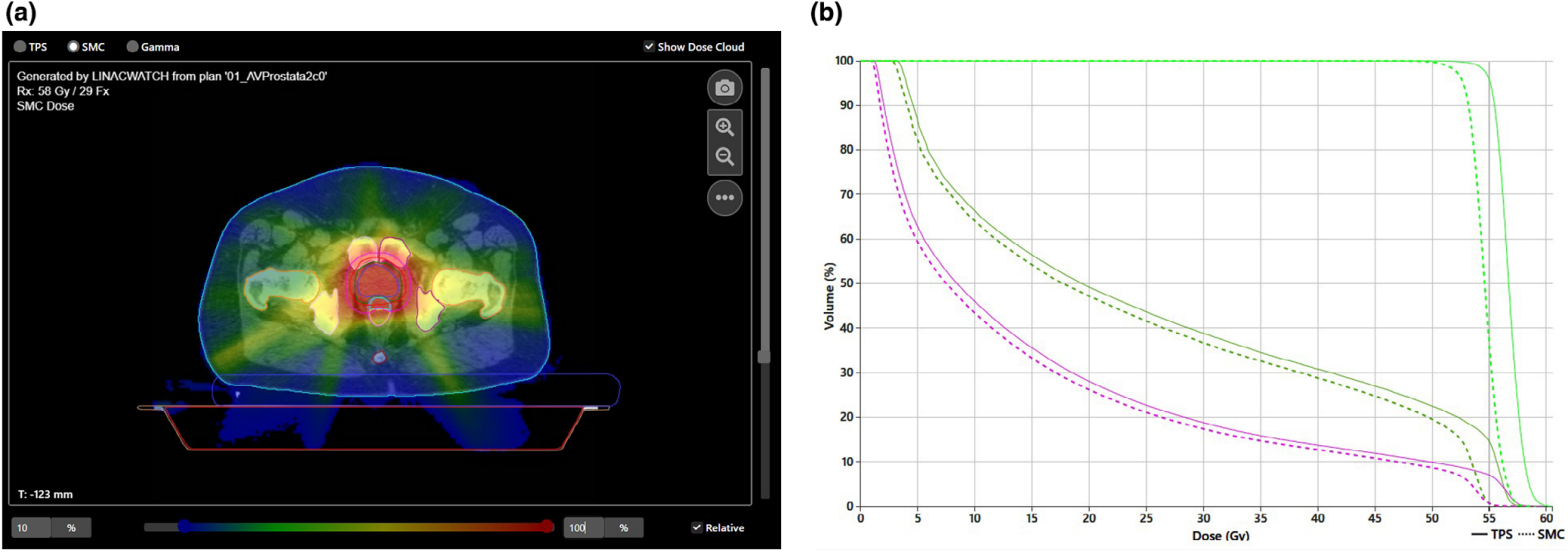
On‐treatment QA of the error induced plan (MLC shift error −0.75mm). (a) SciMoCa dose distribution of MLC error induced plan with −0.75 mm MLC shift (RT log file plan). (b) DVH analysis between TPS and SciMoCa dose distribution (dark green line: bladder, purple line: rectum, light green line: PTV).

Further details for MLC shift error are extracted from dose distribution and DVH analysis in SciMoCa. Figure 7 shows the dose distribution and DVH analysis of on‐treatment QA for MLC shift error of −0.75 mm.

## 4 DISCUSSION

Using 4‐Hz log files with an independent secondary Monte Carlo dose calculation algorithm enables on‐treatment QA for every fraction. When using log files for patient‐specific QA, discrepancies between the original Monaco RT plan, which drives the linac, and the RT log file plan generated by the linac are not acceptable. Thus, calculations with both RT plans (Monaco RT plan and RT log file plan) must lead to the same results applying the same calculation method. This is a fundamental requirement to use log files in patient‐specific QA. In this study, all PTV metrics (see Fig. 4 and Table 1) yielded the same results for RT plan and RT log file plan with 4‐Hz log files using SciMoCa. Furthermore, the GPR calculations for pretreatment and on‐treatment patient‐specific QA are consistent (see Table 2). If there were disagreements of GPR calculations or other PTV metrics between pretreatment and on‐treatment QA, a more precise DVH comparison (including all clinically relevant dose regions) and further investigation would be required to find the source of the error or the cause of the deviation. The study showed significant discrepancies for OARs rectum and bladder between pretreatment and on‐treatment QA. Possible reasons for these discrepancies are the usage of two independent base data sets and the different implementation of the MC algorithms (TPS and SciMoCa). Moreover, small deviations between RT plan and RT log file plan can occur due to dynamic tolerances of linac delivery or sampling the delivery with 4 Hz only. Since these OARs are low dose regions, larger relative errors occur but their absolute dose deviations are negligible.

Consistent results between TPS Monaco, SciMoCa and linac delivery were obtained with ionization chamber measurements. The agreement of pretreatment and on‐treatment patient‐specific QA demonstrated that TPS Monaco and SciMoCa were well implemented in our clinical routine.

Heilemann et al. investigated the sensitivity of MLC shift errors for a prostate plan using phantom‐based measurements. The influence of MLC shift errors on PTV Dmean was evaluated and deviations from MLC shift errors similar to our findings were described. However, the sensitivity of their measurement‐based QA was inferior to the performance of our on‐treatment QA using log files.

A significant advantage of patient‐specific QA with log files is that the influence of MLC shift errors on PTV Dmean can directly be seen in the patient’s dose distribution in SciMoCa. In measurement‐based QA, PTV Dmean cannot be assessed.

In comparison to phantom‐based QA measurements, using log files is very time‐saving (calculation time of just about 5 min per fraction using MC) and much more efficient in finding errors.^15^, ^16^ Furthermore, log file analysis with simple fluence calculation outperforms ArcCHECK (3D Array, Sun Nuclear) measurements due to sensitivity for VMAT plans.^17^ The advantage of using an independent MC algorithm instead of a simple fluence calculation is its accuracy of dose calculation (gold standard in clinical routine). Therefore, dose calculation errors of TPS and errors in base data of TPS can be identified. The impact of all introduced errors on dose distribution in patient's CT is calculated directly and can instantly be used for further investigations. Combining log files and MC also enables identifying errors in plan transfer to the linac and delivery of the linac. A combination of log files and MC requires precise commissioning of the beam model and well‐established machine QA to ensure high treatment quality.

In general, all recorded log files are insensitive to miscalibration.^16^, ^38^, ^39^ Accuracy of log files is crucial for patient‐specific QA. Norvill et al. investigated the clinical impact of multi‐leaf collimator (MLC) calibration errors and showed that the mean PTV dose followed a linear trend with MLC error, increasing at rates of 3.2%–5.9% per millimeter depending on treatment site. Therefore, MLC accuracy of calibration is important when applying modulated radiotherapy delivery techniques.^14^ McKenzie et al. demonstrated that commonly used measurement devices perform poorly in identifying unacceptable plans.^40^

There are limitations to this study, since only prostate plans were analyzed. Moreover, every calculation was performed with the planning CT instead of the daily CT at the linac and no changes in anatomy or patient position were taken into consideration.^41^ The next step is to include the daily CT into our described on‐treatment patient‐specific QA program to see dose distribution of the day in the actual anatomy of the patient.

## 5 CONCLUSION

Our study demonstrates that 4‐Hz log files and an independent secondary MC dose calculation algorithm have the potential to replace or reduce time‐consuming and labor‐intensive phantom‐based measurements as patient‐specific QA. The SciMoCa calculations of the Monaco RT plans and the RT log file plans are in good agreement to each other, thus, the 4‐Hz log files are a suitable method for checking TPS calculation, plan transfer to the linac and delivery/treatment itself.

## CONFLICTS OF INTEREST

The authors have no relevant conflicts of interest to disclose.

## AUTHOR CONTRIBUTIONS

Study conception and design: Philipp Szeverinski, Matthias Kowatsch, Thomas Künzler, Marco Meinschad, Patrick Clemens and Alexander F. DeVries.

Data collection, analytic calculations, statistics and interpretation: Philipp Szeverinski, Matthias Kowatsch, Thomas Künzler.

Draft manuscript: Philipp Szeverinski.

All authors provided critical feedback for analysis and text in the manuscript. All authors reviewed the manuscript and accepted the final version.

## Data Availability

The data that support the findings of this study are available from the corresponding author upon reasonable request.
